# *Mycoplasma pneumoniae* detections in children with lower respiratory infection before and during the COVID-19 pandemic: a large sample study in China from 2019 to 2022

**DOI:** 10.1186/s12879-024-09438-2

**Published:** 2024-06-01

**Authors:** Weiling Qiu, Jiaying Ding, Hongmei Zhang, Shumin Huang, Zuowei Huang, Ming Lin, Yuanyuan Zhang, Zhimin Chen

**Affiliations:** Department of Pulmonology, Children’s Hospital, Zhejiang University School of Medicine, National Clinical Research Center for Child Health, National Children’s Regional Medical Center, No.3333 Binsheng Road, Zhejiang, Hangzhou, People’s Republic of China

**Keywords:** Mycoplasma pneumoniae, COVID-19, Lower respiratory tract infection, Non-pharmaceutical interventions, Epidemiological trend

## Abstract

**Background:**

Nonpharmaceutical interventions (NPIs) implemented to reduce the transmission of severe acute respiratory syndrome coronavirus 2 (SARS-CoV-2) have suppressed the spread of other respiratory viruses during the coronavirus disease 2019 (COVID-19) pandemic. This study aimed to explore the epidemiological trends and clinical characteristics of *Mycoplasma pneumoniae* (MP) infection among inpatient children with lower respiratory tract infection (LRTI) before and during the COVID-19 pandemic, and investigate the long-term effects of China’s NPIs against COVID-19 on the epidemiology of MP among inpatient children with LRTI.

**Methods:**

Children hospitalised for LRTI at the Department of Pulmonology, The Children’s Hospital, Zhejiang University School of Medicine (Hangzhou, China) between January 2019 and December 2022 were tested for common respiratory pathogens, including *Mycoplasma pneumoniae* (MP), *Chlamydia trachomatis* (CT) and other bacteria. Clinical data on age, sex, season of onset, disease spectrum, and combined infection in children with MP-induced LRTI in the past 4 years were collected and analysed.

**Results:**

Overall, 15909 patients were enrolled, and MP-positive cases were 1971 (34.0%), 73 (2.4%), 176 (5.8%), and 952 (20.6%) in 2019, 2020, 2021, and 2022, respectively, with a significant statistical difference in the MP-positive rate over the 4 years (*p* <0.001). The median age of these children was preschool age (3–6 years), except for 2022, when they were school age (7–12 years), with statistical differences. Comparing the positive rates of different age groups, the school-age children (7–12 years) had the highest positive rate, followed by the preschoolers (3–6 years) in each of the 4 years. Compared among different seasons, the positive rate of MP in children with LRTI was higher in summer and autumn, whereas in 2020, it was highest in spring. The monthly positive rate peaked in July 2019, remained low from 2020 to 2021, and rebounded until 2022. Regarding the disease spectrum, severe pneumonia accounted for the highest proportion (46.3%) pre-pandemic and lowest (0%) in 2020.

**Conclusion:**

Trends in MP detection in children with LRTIs suggest a possible correlation between COVID-19 NPIs and significantly reduced detection rates. The positivity rate of MP gradually rose after 2 years. The epidemic season showed some differences, but school-age children were more susceptible to MP before and during the COVID-19 pandemic.

## Background

*Mycoplasma pneumoniae* (MP) can be transmitted via respiratory droplets, causing respiratory tract infections [1]. It stands as one of the most prevalent pathogens causing respiratory diseases in children, accounting for up to 40% of community-acquired pneumonia cases in the paediatric population [2]. This percentage tends to increase during epidemics. While MP infections are generally self-limiting, they can lead to refractory pneumonia and extrapulmonary injury, resulting in severe complications and even death. The MP epidemic has had a serious impact on children, society, and medical resources. The periodic occurrence of MP epidemic, with intervals of 3–7 years and durations of up to 2 years, has been observed. It was estimated that 2019–2020 would be another year of MP epidemic [3]. The coronavirus disease 2019 (COVID-19) pandemic has significantly altered the epidemiology of other viral respiratory infections [4, 5]. Investigating the impact of the COVID-19 pandemic on the prevalence of MP is crucial.

During the COVID-19 pandemic, non-pharmaceutical interventions (NPIs) were proposed to mitigate the transmission of severe acute respiratory syndrome coronavirus 2 (SARS-CoV-2) [6]. Since the onset of the pandemic, the Chinese government had implemented extensive and strict NPIs, including mask-wearing, social distancing, home-based work, and school closures. In the first half of 2020, most cities in China, including Hangzhou in Zhejiang Province, enforced strict NPIs. Hangzhou, being the first province to initiate a “first-level emergency plan” on 23 January, responded with a comprehensive set of measures for a significant public health emergency. These measures included working from home, closing public cultural or entertainment places, and implementing work from home policies until gradual reopening of classes at the end of April. From then until the end of December 2022, “relaxed NPIs”, including mask-wearing and physical-distancing, were mandated in public areas. If SARS-CoV-2 positive cases were reported during this period, the specific location was considered high-risk, triggering the enforcement of strict NPIs. Temporary suppression of influenza epidemics and other viral respiratory diseases, including the respiratory syncytial virus (RSV), has been associated with the global implementation of NPIs [7–9]. The three-year comprehensive control measures implemented in China differ significantly from those in other countries, warranting a study on their impact on the epidemic trend of respiratory pathogens.

Recent studies have focused on MP trends before and during the pandemic in China [10, 11]; however, these results were obtained using pre-pandemic data, and lacked data from the later stages of the epidemic. In the present study, we conducted a retrospective epidemiologic analysis of MP prevalence during the last four years to explore the characteristics of inpatient children with MP in Hangzhou, and to investigate the effect of the COVID-19 pandemic on the prevalence of MP.

## Methods

### Study design

Data were retrospectively analysed for paediatric in-patients between 1 month and 18 years of age with LRTI who were admitted to the respiratory ward of the Children’s Hospital of Zhejiang University School of Medicine between 1 January 2019 and 31 December 2022. All children were diagnosed with LRTI, manifesting fever and/or lower respiratory tract symptoms, such as tachypnoea, non-productive cough, wheezing, and increased breath sounds. Additionally, each in-patient exhibited at least one of the following conditions indicative of LRTI: rales or wheezes, or chest imaging findings, encompassing diagnoses such as pneumonia, bronchitis, bronchiolitis, and prolonged pneumonia. Respiratory pathogens were analysed in all enrolled patients. Furthermore, the prevalence trends of other pathogens, including RSV [12], were analysed using similar methods. The clinical data of MP-positive children, including age, sex, length of stay (LOS), seasonal distribution, co-infecting pathogens, and diagnosis, were comprehensively analysed to evaluate the impact of the pandemic. A comparison of data for four consecutive years from 2019 to 2022 reflected the epidemiological trend of MP detection after the COVID-19 outbreak.

### Measurement

Respiratory specimens (pharyngeal swabs or nasopharyngeal aspirates) were collected from the enrolled in-patients with LRTI by trained staff in accordance with the standard operating procedures. All specimens were tested by direct immunofluorescence (DIF) test for RSV, influenza virus A (Flu A), influenza virus B (Flu B), adenovirus (ADV), para-influenza virus 1 (PIV-1), para-influenza virus 2 (PIV-2), para-influenza virus 3 (PIV-3). MP and *Chlamydia trachomatis* (CT) were tested for by polymerase chain reaction (PCR), and cultures were grown to detect bacteria. If MP was detected, the patient was enrolled for further analysis. Co-infection was evaluated according to the test results for other pathogens.

### Data processing and statistical analyses

Categorical variables were expressed as ratios or numbers (%). The age and LOS of the patients were expressed as medians and interquartile ranges (IQR) as they were not normally distributed. χ2 and rank-sum tests were used when comparing respective groups. A *p* value below 0.05 was considered statistically significant. All data were analysed using the SPSS software package (version 24.0, IBM, Armonk, NY, USA).

## Results

### Patient characteristics

Between January 2019 and December 2022, 15909 children were admitted to the pulmonary department due to LRTI: 5271 cases in 2019, 3006 cases in 2020, 3013 cases in 2021, and 4619 cases in 2022. Among all the enrolled patients, MP-positive cases and proportions were 1791 (34.0%), 73 (2.4%), 176 (5.8%), and 952 (20.6 %) in 2019, 2020, 2021, and 2022, respectively, with a significant statistical difference in the MP-positive rate over the 4 years (*p* <0.001) and between every 2 years. The monthly positive rate of children with MP and LRTI peaked in July 2019 (61.7%), while the highest monthly positive rate annually from 2020 to 2022 was in April (9.2%), August (16.9%), and November (31.4%), respectively. The characteristics of LRTI with MP detection between 2019 and 2022 are shown in Table 1. No significant difference was observed in the sex ratio of MP detected over 4 years (*p* = 0.488). The median age of patients in the preschool or school-age group was 55, 61, 54, and 84 months in 2019, 2020, 2021, and 2022, respectively, and the median age in 2022 was higher than that in the other 3 years (*p* <0.001). The median LOS in 2022 (5 d) was shorter than that in the other 3 years (*p* <0.001). Co-detection proportion in 4 years was 10.2–16.4%, with no statistical difference (*p* = 0.219). Regarding the disease spectrum, all children with LRTI and MP were classified as having acute pneumonia or acute bronchopneumonia, acute bronchitis, prolonged pneumonia, bronchiolitis, and severe pneumonia. Within 4 years, a statistically significant difference was observed in the distribution of each disease (*p* <0.001). In 2019, pre-COVID-19 pandemic, the proportion of severe pneumonia was the highest, with statistical differences compared to the other 3 years, and lowest in 2020, which was also statistically different from the other 3 years. The number of cases of co-detection with other pathogens was 282 (15.7%), 15 (20.5%), 18 (10.2%), and 87 (8.9%) in 2019, 2020, 2021, and 2022, respectively, with a statistically significant difference (*p* <0.001). Statistical analysis was performed to determine whether MP-positive cases were combined with the other seven respiratory viruses and CT and whether the bacterial culture was positive. The proportion of co-detections with other pathogens in 2019 and 2020 was relatively high, whereas that in 2022 was relatively low. Post hoc analysis revealed statistically significant differences between 2019 and 2022, 2020 and 2021, and 2020 and 2022. Among the co-infecting pathogens, ADV accounted for the most significant proportion in 2019 and 2022, whereas RSV and bacteria were most common in 2020 and 2021, respectively.

**Table 1:** Characteristics of LRTI with MP detections from  2019 to 2022

**Characteristics**	**2019(*n*=1791)**	**2020(*n*=73)**	**2021(*n*=176)**	**2022(*n*=952)**	***P* value**
Ratio of MP detections to total of patients with LRTI	34.0% (1791/5271)^b,c,d^	2.4% (73/3006)^a,c,d^	5.8% (176/3013)^a,b,d^	20.6% (952/4619)^a,b,c^	<0.001
Age (month), median(IQR)	55.0(30.0, 84.0)^d^	61.0(28.0, 72.0)^d^	54.0(24.0, 84.0)^d^	84.0(53.5, 96.0)^a,b,c^	<0.001
Gender (male: female)	1.1:1	1.0:1	1.4:1	1.4:1	0.488
LOS (day), median (IQR)	7.0(5.0, 9.0)^d^	6.0(5.0, 9.0)^d^	6.0(5.0, 8.0)^d^	5.0(4.0, 7.0)^a,b,c^	<0.001
LRTI diagnosis, n (%)					<0.001
Acute pneumonia or acute bronchopneumonia, n (%)	898(50.2%)^b,c,d^	50(68.5%)^a^	108(61.4%)^a^	564(59.2%)^a^	
Acute bronchitis, n (%)	11(0.6%)^b^	5(6.8%)^a,c,d^	2 (1.1%)^b^	5(0.5%)^b^	
Prolonged pneumonia, n (%)	44(2.5%)^b^	18(24.7%)^a,c,d^	3(1.7%)^b^	16(1.7%)^b^	
Bronchiolitis, n (%)	9(0.5%)	0(0%)	2(1.1%)	2(0.2%)	
Severe pneumonia, n (%)	829(46.3%)^b,c,d^	0(0%)^a,c,d^	61(34.7%)^a,b^	365(38.3%)^a,b^	
Co-infection, n(%)	282(15.7%)^d^	15(20.5%)^c,d^	18(10.2%)^b^	87(8.9%)^a,b^	<0.001
Flu	15(5.3%)	2(2.7%)	1(0.6%)	9(0.9%)	
ADV	109(38.7%)	1(1.4%)	1(0.6%)	32(3.4%)	
PIV	72(25.5%)	0(0%)	1(0.6%)	14(1.4%)	
RSV	14(5.0%)	6(8.2%)	5(2.8%)	21(2.2%)	
CT	4(1.4%)	0(0%)	1(0.6%)	0(0%)	
Bacteria	68(24.1%)	6(8.2%)	9(5.1%)	11(1.2%)	

^a^represents *P*<0.05 compared to group 2019

^b^represents *P*<0.05 compared to group 2020

^c^represents *P*<0.05 compared to group 2021

^d^represents *P*<0.05 compared to group 2022

### Age stratification

**Fig. 1:**
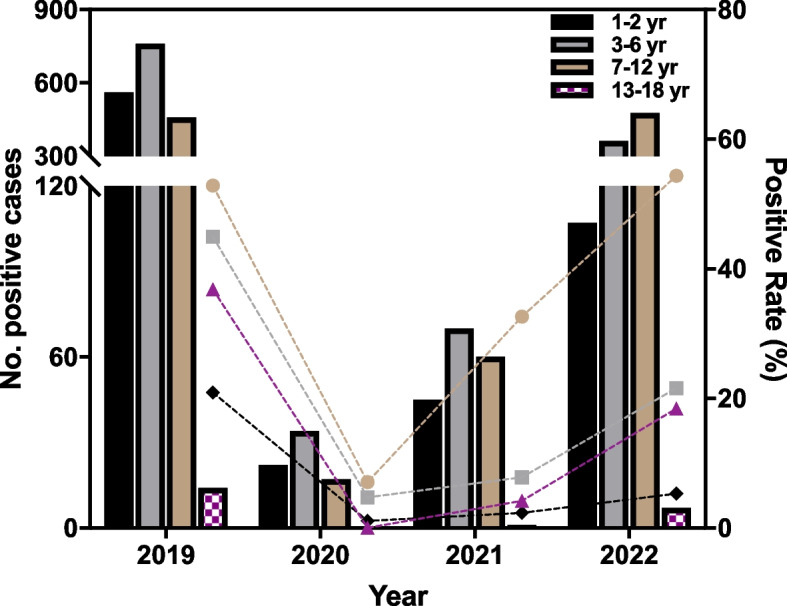
MP-positive rates and number of positive cases among different age groups from 2019 to 2022

The patients were divided into four groups according to age: infants (1–2 years), pre-schoolers (3–6 years), school-aged children (7–12 years), and adolescents (13–18 years). The MP positive rates and the number of MP positive cases in different age groups from 2019 to 2022 are shown in Table 2 and Fig. 1. The positive rates of the various groups showed that school-age children had the highest positive rate annually, followed by the preschoolers. Except in 2020, MP was not detected in adolescents, and the lowest MP-positive rate was observed in infants in the other 3 years. The positive rates of the different age groups annually and those of various years in each age group were significantly different (*p* <0.001).

**Table 2:** Positive rate (%) of MP in different age groups of children with LRTI

**Age groups(y)**	**2019**	**2020**	**2021**	**2022**	**x**^**2**^	***P***^**a**^**- value**
≤2	20.9(560/2677)	1.1(22/2008)	2.4(45/1911)	5.3(107/2028)	791.4	<0.001
3-6	44.9(759/1689)	4.7(34/719)	7.8(70/894)	21.6(362/1677)	673.4	<0.001
7-12	52.8(458/867)	7.1(17/240)	32.6(60/184)	54.3(476/876)	199.6	<0.001
13-18	36.8(14/38)	0(0/39)	4.2(1/24)	18.4(7/38)	22.5	<0.001
**x**^**2**^	431.4	54.0	288.5	901.5	—	—
***P***^**b**^**-value**	<0.001	<0.001	<0.001	<0.001	—	—

P^a^ refers to comparing of the positive rates of each age group in different years

P^b^ refers to the comparison of the positive rates of each year in the different age groups

### Seasonal distribution

**Fig. 2:**
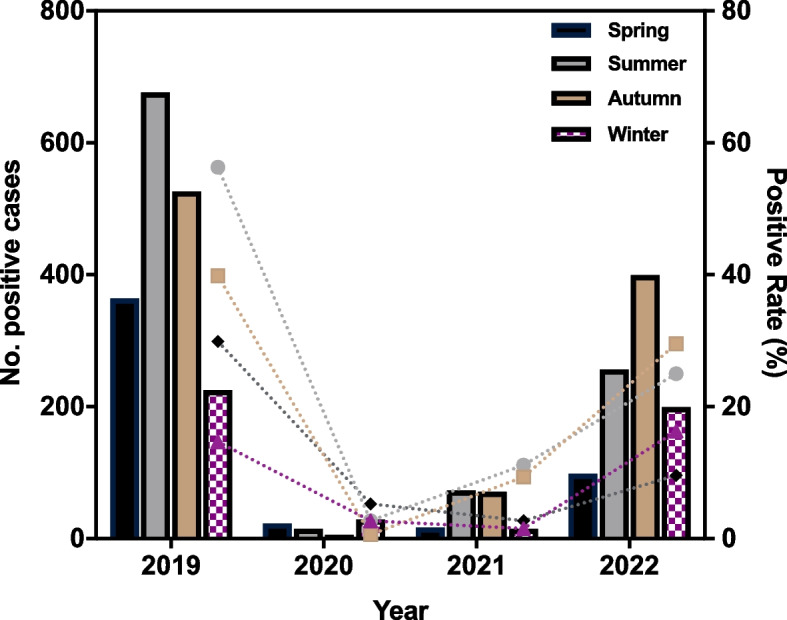
MP-positive rates and number of positive cases among different seasons from 2019 to 2022

Patients were categorized into four admission periods based on their admission dates: Winter, Spring, Summer, and Autumn. Winter was defined as December to February, spring as March to May, summer as June to August, and autumn as September to November. The positivity rates of MP stratified by season in children with LRTI are shown in Table 3 and Fig. 2. Comparison of the MP-positive rates of different seasons in 4 years revealed a statistically significant difference (*p* <0.001). The incidence was highest in summer, followed by autumn in 2019 and 2021, while it was highest in spring at the beginning of the COVID-19 pandemic in 2020, and in 2022, the incidence was highest in autumn, followed by summer, with statistical differences compared with the distribution in 2019, the year prior to the pandemic.

**Table 3:** Positive rate (%) of MP stratified by season in children with LRTI

**Seasons**	**2019**	**2020**	**2021**	**2022**	**x**^**2**^	***P***^**a**^**-value**
**Winter (Dec.-Feb.)**	14.7(225/1532)	2.7(29/1076)	1.5(15/978)	16.3(199/1223)	237.8	<0.001
**Spring (Mar.-May.)**	29.9(364/1217)	5.3(23/436)	2.7(17/622)	9.6(98/1021)	336.9	<0.001
**Summer (Jun.-Aug.)**	56.3(676/1201)	2.7(15/557)	11.2(73/654)	25.0(256/1023)	719.6	<0.001
**Autumn (Sep.-Nov.)**	39.8(526/1321)	0.6(6/937)	9.4(71/759)	29.5(399/1352)	588.8	<0.001
**x**^**2**^	549.6	28.0	94.6	167.3	—	—
***P***^**b**^**-value**	<0.001	<0.001	<0.001	<0.001	—	—

P^a^refers to comparing of the positive rates for each season in different years

P^b^ refers to the comparison of the positive rates for each year in different seasons

**Fig. 3:**
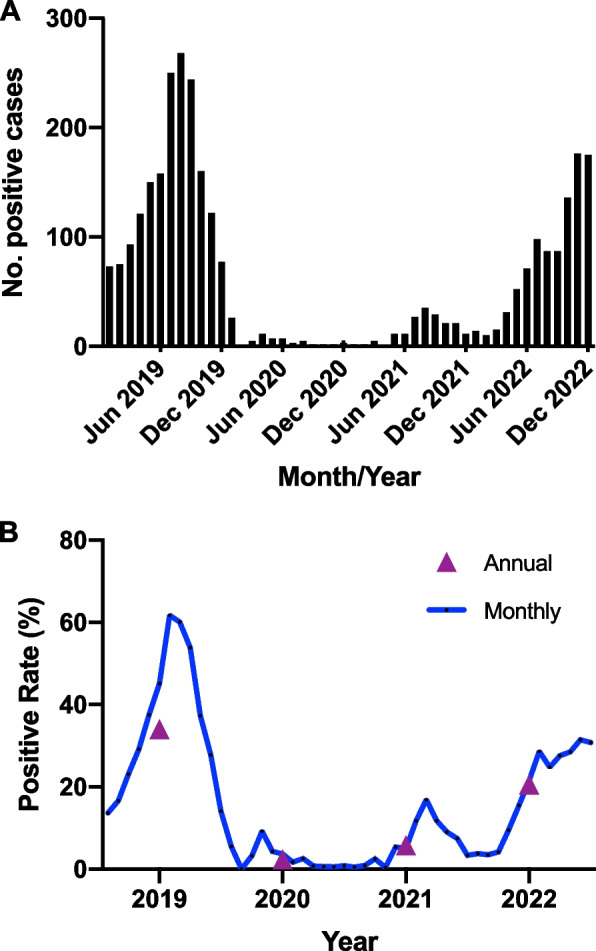
MP-positive cases monthly (**A**) and MP-positive rates monthly and yearly (**B**) from 2019 to 2022

Based on the monthly distribution of MP-positive cases over 4 years, the number of cases gradually increased from the beginning of 2019, peaked in August, remained high during the summer, then gradually decreased, and remained low from 2020 to 2021. By 2022, the number of cases will rebound and peak by the end of 2022. This epidemiological trend of MP can also be seen in the monthly positive rate over the 4 years (Fig. 3).

## Discussion

This study investigated the clinical features and epidemiological trends of MP infections before and during the COVID-19 pandemic. Additionally, the analysis of 4-year clinical data showed an epidemiological trend under various preventive and control measures. Epidemic prevention strategies vary across nations and areas and may affect MP prevalence differently. Our findings indicated the impact of these strategies on MP prevalence in Southeast China. Mitigating the transmission of SARS-CoV-2 requires drastic adjustments to population-level community behaviour, affecting the spread of endemic respiratory diseases [13]. Both stringent and relaxed NPIs may have reduced the spread of respiratory viruses, including SARS-CoV-2, RSV, and the influenza virus [13, 14]. However, studies have also indicated that viral respiratory infections have been common during the COVID-19 pandemic. For instance, our previous research revealed that RSV was the primary causative agent of LRTI during the pandemic, and variations in the trends of susceptible populations and epidemic seasons were observed before and during the pandemic [12].

Different pathogens transmitted via respiratory droplets may exhibit different trends in prevalence during the COVID-19 pandemic. As the primary causative pathogen of community-acquired pneumonia in children, MP remains a concern for pediatricians. This study aimed to understand the prevalence and clinical characteristics of hospitalized children with MP infection during the pandemic. This large sample study of 4 consecutive years summarizes the changes in the seasonal distribution of epidemics. It clarifies the demographic characteristics and disease severity in hospitalized children with MP-induced LRTI. Our study comprehensively investigated MP infection’s prevalence and clinical characteristics in children before and during the pandemic. It provided information for prevention and treatment strategies for the future mitigation of MP infection in children.

Our study found that among hospitalized children with LRTI, the detection rate of MP was highest in 2019, decreased to the lowest in 2020, and recovered to 20.6% by 2022, with statistical differences. This reflects the epidemiological trend of MP detection for four consecutive years before and during the COVID-19 pandemic. According to the seasonal and monthly MP-positivity rates, a peak was observed in the summer of 2019, similar to previous studies [10, 11]. Based on past trends, a recent MP outbreak was predicted to extend from the summer and fall of 2019 to the winter or spring of 2021 [15]. However, compared to 2019, the positive instances of MP in 2020 and 2021 were significantly lower. Our findings also indicated a significant decline in MP detection starting in February 2020, which was maintained at a low level with slight oscillations. The number of positive cases steadily increased until April 2022 and persisted until the conclusion of the investigation. Effective COVID-19 pandemic control strategies have the potential to significantly lower MP prevalence in closed or semi-closed populations, including hospitals, schools, religious centers, and military bases [16–18]. The significant decline in the MP-positivity rate observed in 2020 is consistent with previous research [19], and investigations conducted in Finland and Japan showed that MP prevalence was significantly lower in 2020 than in 2012 and 2016 [20, 21]. Stringent protocols to mitigate the spread of SARS-CoV-2 might have affected the spread of other respiratory viruses with similar transmission modes. NPIs such as obligatory facemasks, social isolation, stay-at-home orders, and hand hygiene were primary global initiatives. All these measures aided in reducing the spread of MP via droplets and contact with unclean hands and feet. Our research also found that although it was during the COVID-19 pandemic, MP-positive cases gradually increased until 2022. By the end of 2022, the number of positive instances exceeded the level of the same period in 2019. This may be related to the implementation of relaxed NPIs in Hangzhou in 2022, where children attend school and participate in activities normally; However, after 2 years of low infection proportion with MP, the population lacks immune protection and is generally susceptible to MP, resulting in a gradual increase in the number of positive cases.

From the perspective of age stratification, most positive cases were observed among preschool and school-aged children, consistent with the characteristics of MP infections in children [14]. Although the annual positivity rate varied greatly from the perspective of age distribution, the positivity rate of school-age children was the highest both before and during COVID-19. In 2022, the MP-positivity rate of school-aged children reached 54.3%, and the median age was higher than that of the other 3 years, indicating statistical differences. In 2022, the first year during the epidemic in which the infection rate of MP had gradually increased after a sustained low level of infection, most cases were among school-age children, suggesting that school-age children are more susceptible to MP infection in a population with a generally decreased immune protection against it. Compared with infants, the safety of school-aged children is easily neglected, rendering them more susceptible to MP infection. However, this requires further investigation.

Regarding disease spectrum and severity, acute pneumonia dominated, accounting for 50.2–68.5% of MP-positive cases; moreover, the proportion of severe pneumonia was high. Except for 2020, a year with a meagre MP detection rate, the proportion of severe pneumonia in other years was above 30%; suggesting that MP is an important pathogen of community-acquired pneumonia in children and an important pathogen that causes severe pneumonia in children. In our study, 8.9–20.5% of children were co-detected with other pathogens within 4 years. The co-detection rate was relatively low in 2021 and 2022, possibly related to the selection of pathogens detected in this study. Common respiratory viruses, such as rhinovirus, coronavirus, and metapneumovirus, were not detected. The co-detection rate was also related to the prevalence of other viruses. For instance, more mixed adenovirus infections in 2019 were associated with the prevalence of adenoviruses in 2019 [22].

Compared with previous studies [10, 15], the nucleic acid test was performed to investigate the MP-positivity rate in our research, which was more reliable than specific IgM antibodies for diagnosing MP infection, as the time and duration of antibody production may influence IgM antibody test results. In addition, our study period, which lasted from before the end of the pandemic, was meaningful for a comprehensive understanding of MP prevalence during the entire epidemic period. Our study has some limitations. First, in the first 2 years of the pandemic, especially in early 2020, when the NPIs were strictly implemented, the total number of cases of LRTI for admission was low, which may have been related to delays in patients’ visits to hospital emergency emergencies and face-to-face outpatient clinics, which may have created a selection bias. Second, this was a single-center study, and it would have been more convincing if the data had been obtained from different centers. In addition, our research should have covered the period after China implemented prevention and control measures for category A infectious diseases against SARS-CoV-2 infection. Therefore, it is not yet possible to determine MP prevalence after the end of the pandemic. Further research is required to determine whether MP prevalence will return to pre-COVID-19 levels by the end of the pandemic.

## Conclusion

Through a study on MP prevalence during NPI enforcement during the COVID-19 pandemic, we compared the data before and during the epidemic for four consecutive years. We found that MP-positive cases sharply decreased at the beginning of the COVID-19 outbreak and gradually rebounded after 2 years, indicating a possible correlation between COVID-19 NPIs and significantly reduced detection numbers. By comparing the seasonal and age distribution of MP-positive rates, it was concluded that MP-positive cases gradually recovered after 2 years of NPI implementation or relaxed NPIs, and the epidemic season showed some differences, while school-age children were all more susceptible to MP both before and during the COVID-19 pandemic.

## Data Availability

The datasets used and analyzed during the current study are available from the corresponding author on reasonable request.

## Abbreviations

NPIs: Non-pharmaceutical interventions
SARS-CoV-2: Severe acute respiratory syndrome coronavirus 2
MP: *Mycoplasma pneumonia*
COVID-19: The coronavirus disease 2019
LRTI: Lower respiratory tract infection
CT: *Chlamydia trachomatis*
RSV: Respiratory syncytial virus
LOS: Length of stay
DIF: Direct immunofluorescence
Flu A: Influenza virus A
Flu B: Influenza virus B
ADV: Adenovirus
PIV-1: Para-influenza virus 1
PIV-2: Para-influenza virus 2
PIV-3: Para-influenza virus 3
PCR: Polymerase chain reaction
IQR: Interquartile ranges
